# Over-expression of NOTCH1 as a biomarker for invasive breast ductal carcinoma

**DOI:** 10.1007/s13205-016-0373-2

**Published:** 2016-02-13

**Authors:** Mahdi Paryan, Rezvan Tavakoli, Seyed Mohammad Ali Hosseini Rad, Neda Feizi, Fereshteh Kamani, Ehsan Mostafavi, Samira Mohammadi-Yeganeh

**Affiliations:** 1 Department of Research and Development, Production and Research Complex, Pasteur Institute of Iran, Tehran, Iran; 2 Department of Molecular Biology and Genetic Engineering, Stem Cell Technology Research Center, Tehran, Iran; 3 Laboratory of Influenza Research, Pasteur Institute of Iran, Tehran, Iran; 4 Department of Surgery, Taleghani Hospital, Shahid Beheshti University of Medical Sciences, Tehran, Iran; 5 Department of Epidemiology, Pasteur Institute of Iran, Tehran, Iran; 6 Cellular and Molecular Biology Research Center, Shahid Beheshti University of Medical Sciences, Tehran, Iran; 7 Department of Biotechnology, School of Advanced Technologies in Medicine, Taleghani Hospital, Shahid Beheshti University of Medical Sciences, Velenjak, Shahid Chamran Freeway, Tehran, Iran

**Keywords:** Breast cancer, NOTCH1, Invasive ductal carcinoma, p53

## Abstract

Breast cancer is the leading cause of cancer-related death in women worldwide. Invasive ductal carcinoma (IDC) is the most frequent invasive form of breast cancer followed by metastasis. There is no accepted marker for distinguishing this form from other less aggressive forms of breast cancer. Therefore, finding new markers especially molecularly detectable ones are noteworthy. It has been shown that NOTCH1 has been overexpressed in the patients with breast cancer, but no study has investigated the expression of NOTCH1 and its correlation with other molecular and hormonal markers of breast cancer so far. In the current study, 20 breast cancer tissues and 20 matched adjacent normal breast tissue from breast cancer patients were obtained and categorized in two groups: patients with IDC and patient with other types of breast cancer. Gene expression analysis using real-time PCR showed that the NOTCH1 gene was significantly overexpressed in patients with IDC. We also found a slight correlation between NOTCH1 overexpression and p53 accumulation in the cancerous cells confirmed by Immunohistochemistry (IHC). This results showed that it is possible to introduce NOTCH1 expression as a novel biomarker of IDC, alone or preferably accompanied by IHC of p53. We also can design new therapeutic agents targeting NOTCH1 expression for inhibition of metastasis in ductal breast carcinoma.

## Introduction

Breast cancer is the most common malignancy in women worldwide. Despite the improvements in therapies for breast cancer such as chemotherapy, radiotherapy and hormone therapy, recurrent rates are high (Weigelt et al. 2005; Rad et al. 2015). Like other malignancies, two important factors that make a treatment successful in breast cancer are early diagnosis and prognosis (Turner et al. 2014). There is always a lacuna in the diagnosis of breast cancer at early stages (Donepudi Ms Fau-Kondapalli et al. 2014). Specific gene expression signatures have been used to create new tests that could offer better prognosis than the traditional diagnostic methods. Therefore, new molecular markers are needed as prognostic tools in breast cancer.

After surgery, gene expression profile of breast tumor might be an applicable way for identification of patients who are more likely to develop metastasis to distant organs specially bone, lung, and liver. Microarray studies showed a significant difference in gene expression profile of metastatic and in situ localized tumors (Fu et al. 2014; Kumar et al. 2012). Furthermore, gene expression signature of breast tumor may be a useful tool for identification of new therapeutics (Rad et al. 2015). Improving our understanding of molecular mechanisms underlying metastasis and cancer invasiveness will also help clinical decision-making process for patients with cancer.

From different subtypes of invasive and metastatic breast cancers, invasive ductal carcinoma (IDC) has the frequency of 50–80 % in invasive forms (Chen et al. 2014), but there is no accepted marker to distinguish between lesions at high risk from lesions at low risk of developing invasive form. In addition, histological typing is not a powerful marker of metastasis (Reedijk et al. 2005). A large number of putative molecular markers have been reported in different studies, but only a few are applicable for popularization in clinical laboratories (Ko et al. 2013; Yang et al. 2013). In addition to hormone markers such as estrogen receptor (ER), progesterone receptor (PR), and human epidermal growth factor receptor 2 (HER2/neu), the expression of some critical overexpressed genes such as Ki-67 and mutated p53 is routinely determined by immunohistochemistry (IHC) in clinical laboratories for the diagnosis and staging of breast cancer.

Ki-67 is a nuclear protein associated with cell proliferation that is expressed in all proliferative cells and in cell cycle phases except G0 (Cattoretti et al. 1992; Gerdes et al. 1983). Because the expression of Ki-67 in normal breast tissue is low (<3 % of cells), measuring the Ki-67 expression could determine the growth fraction of neoplastic cell populations (Gnant et al. 2011; Inwald et al. 2013; Untch et al. 2013). P53 gene encodes a tumor suppressor protein. Inactivation of Ki-67 leads to overexpression of p53 protein, and its overexpression is commonly observed in breast cancers (Donepudi Ms Fau-Kondapalli et al. 2014; Powell et al. 2014). P53 expression is also used for the prediction of response to chemotherapy or hormone therapy (Soussi and Beroud 2001). Some signaling pathways are also deregulated in breast cancers, and their pattern can be used as a marker for the diagnosis or staging of the disease.

Notch is a key signaling pathway involved in regulation of cell differentiation, proliferation, survival, and maintenance of cancer stem cells (Takebe et al. 2015). Aberrant activation of this pathway has been reported in different cancers including breast cancers (Baker et al. 2014). Reports have showed that high level expression of NOTCH1 was associated with poor survival in primary breast cancer-diagnosed patients (Efstratiadis et al. 2007; Reedijk 2012). Among all Notch receptors, NOTCH1 reveals a dominant expression in cancerous breast tissues (Mittal et al. 2009). Yet, a clear understanding of the role of NOTCH1 as a prognostic marker in different breast cancer types is still lacking. Therefore, in the present study, we hypothesized that NOTCH1 might be a prognostic marker of invasive ductal breast carcinoma.

## Methods and materials

### Samples and cases

Breast cancer tissues and matched adjacent normal breast tissues from breast cancer patients were obtained from 2013 to 2015 from university hospitals in Tehran. Written informed consent for biologic studies was obtained from all patients analyzed in accordance with the Declaration of Helsinki (151th Ethics committee, Shahid Beheshti University of Medical Sciences). Parallel sections were paraffin embedded and prepared for Hematoxylin-eosin (H&E) staining and histological diagnosis. The fresh specimens were stored at 4 °C for 24 h in RNA Later (Qiagen, Germany) and then at −80 °C until further use. None of the patients had undergone prior chemotherapy or radiation therapy. After receiving histological reports, 20 paired samples (10 IDC samples and 10 other types of breast cancer) were selected for further studies.

### RNA extraction, cDNA synthesis

Total RNA was extracted using QIAzol RNA extraction protocol (Qiagen, Germany) according to the manufacturer’s instructions. After confirming the integrity and quality of RNA using spectrophotometer (Eppendorf, Germany), DNaseI treatment was performed according to the manufacturer’s protocol (Sigma-Aldrich, USA). RNA was stored at −80 °C until use. Afterwards, extracted RNAs were reverse-transcribed using random hexamer and Expand Reverse Transcriptase (Roche Diagnostics GmbH Mannheim, Germany) according to the manufacturer’s manual. The cDNA was stored at −20 °C until use.

### Real-time RT-PCR

Triplicate real-time PCR reactions were performed for each gene in a final volume of 13 µl containing 6.25 µl SYBR Premix Ex Taq II (Tli RNase H Plus, Takara, Japan), 0.2 µl forward primer (0.4 µm), 0.2 µl reverse primer (0.4 µm), and 6.35 µl water. Amplification was performed in the following condition: enzyme activation step at 95 °C for 30 s and 40 cycles of two thermal amplification steps including 95 °C for 5 s and 60 °C for 30 s. Post-amplification melting curve analysis was performed by a slow increase in temperature (0.2 °C/s) from 60 °C up to 95 °C. Amplification, data acquisition, and analysis were performed on Rotor-Gene Q Instrument (Qiagen, Germany). Considering that determining crossing point (CP) was necessary for relative gene expression analyses, in this study “second derivate maximum method” was performed for CP determination. Fold change in gene expression was determined using the Relative Expression Software Tool (REST^®^) (Pfaffl et al. 2002). *β*-actin gene was used as the reference gene and underwent all the procedures mentioned earlier. Non-template control and RT-minus were used in all of the experiments. MIQE guidelines were recruited in all of the manipulation steps. Primer sequences are available in Table 1.

**Table 1:** The sequence of primers used in this study

Gene	Accession number	Forward primer	Reverse primer	Product length (bp)
*β*-actin	NM_001101.3	CTTCCTTCCTGGGCATG	GTCTTTGCGGATGTCCAC	86
NOTCH1	NM_017617	CTGGTCAGGGAAATCGTG	TGGGCAGTGGCAGATGTAG	106

### Statistical analysis

Real-time PCR results were analyzed using REST^®^2009 software. SPSS software (version 16.0; SPSS Inc, Chicago, IL) was used to analyze the patients’ results. The distribution status of data was evaluated using Kolmogorov-Smirnov goodness of fit test. Since NOTCH1 expression showed non-normal distribution, non-parametric statistical tests was performed. Group differences in variables were compared using Kruskal–Wallis, Chi-square, and Fisher’s exact test. Graphical procedures were performed using Microsoft Excel 2010.

In all cases, a *p* value less than 0.05 was deemed statistically significant, less than 0.1 was considered slightly significant, and greater than 0.1 was regarded as non-significant.

## Results

Notch signaling pathway is one of the main signaling pathways that afflicts in progression and metastasis of breast cancer. In the present study, we hypothesized that NOTCH1 is up-regulated in IDC.

All the samples retrieved from women undergoing breast cancer surgery. The patients recruited in this study were aged from 30 to 71. Applying histopathology, nine out of twenty samples were confirmed to be ER (+)PR(+)HER2/neu(∓) and others were ER(−)PR(−)HER2/neu (∓). Ten samples which were previously diagnosed as invasive and infiltrating ductal carcinoma were selected. The results are presented in Table 2.

**Table 2:** Investigated parameters of breast cancer samples (*n* = 20)

Characteristics	No # (%)
Age (year)	
≤35	2 (10)
35–50	1 (5)
>51	17 (85)
Nottingham score	
I	2 (10)
II	2 (10)
III	13 (75)
Missing	3 (15)
Histopathological type	
Invasive ductal carcinoma breast cancer	10 (50)
Other Types of breast cancer	10 (50)
p53	
(+)	6 (30)
(−)	8 (40)
Missing	6 (30)
Ki-67	
(+)	16 (80)
(−)	4 (20)

**Fig. 1:**
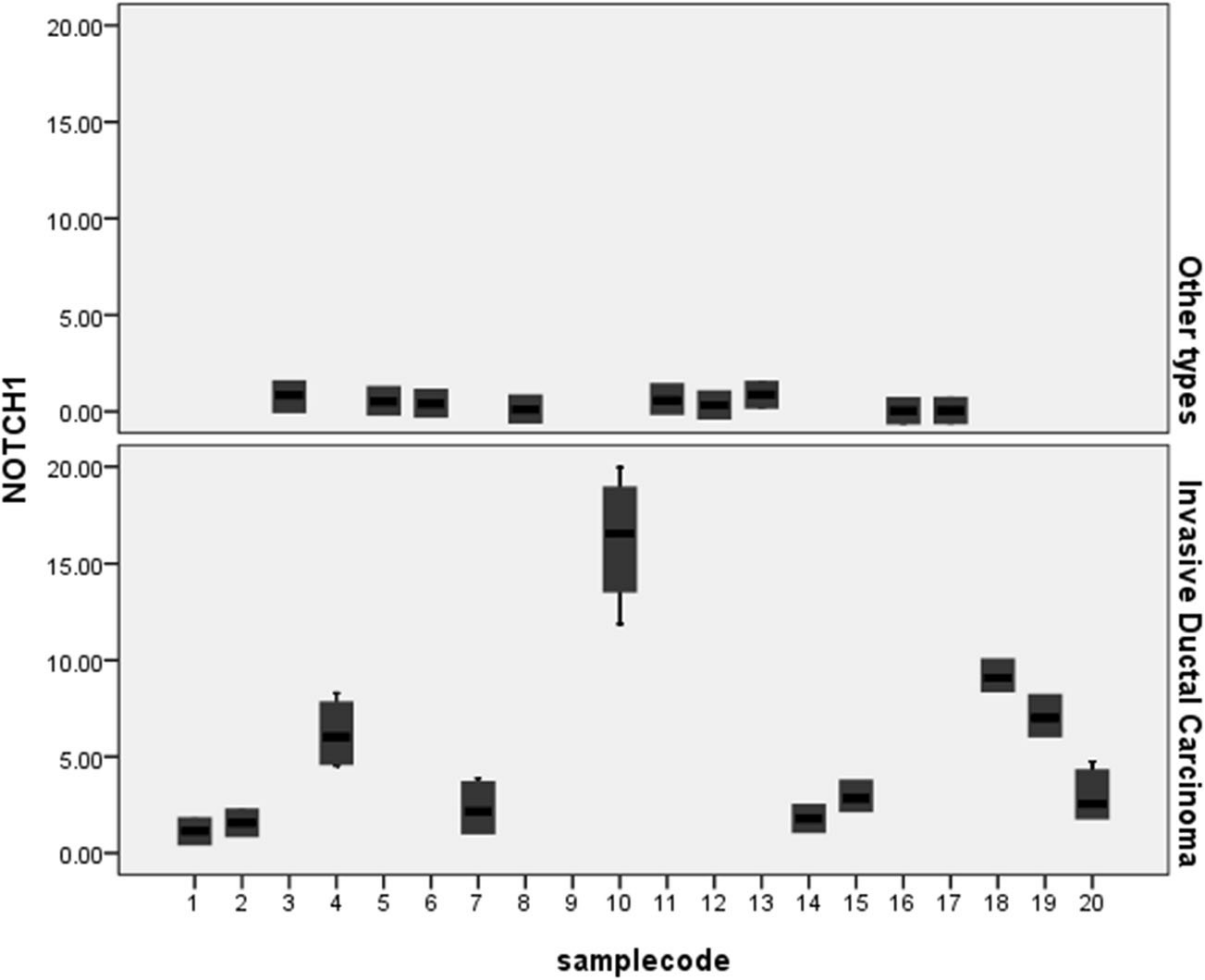
NOTCH1 expression in breast cancer samples. Down, IDC breast cancers. Up, non-IDC (other types)

To test the hypothesis, we recruited a sensitive real-time PCR based on SYBR Green I to evaluate the expression of NOTCH1 in breast cancer and adjacent breast tissue samples. All the primer and reaction setups were performed manually. To find out the expression level of NOTCH1, real-time PCR was carried out for 10 patient samples having ductal carcinoma and 10 patient samples having other types of breast cancer. The expression of NOTCH1 in IDC was significantly higher than that of the patients with other types of breast cancer (*p* value <0.001). In fact, all IDC samples expressed high level of NOTCH1 compared to other types of breast cancers (Fig. 1).

We also investigated the histopathological reports of each sample. Statistical analysis showed that NOTCH1 was slightly correlated with p53 expression (*p* value = 0.091). No significant relation was found between ER, PR, HER2/neu, Ki-67 expression and histopathology of samples or other molecular markers (*p* value >0.1).

## Discussion

Considering the heterogeneity of breast cancer, prediction of invasive or migratory potential of a primary tumor might require determining a lot of biomarkers. All traditional prognostic markers can only identify about 30 % of high-risk patients. Therefore, new molecular markers are needed to help in identifying breast cancer patients who are at high risk of metastasis development and to avoid overtreatment or under treatment of patients. Actually, a promising molecular marker should be able to accurately predict metastatic potential of a breast tumor. Gene-expression profiling may be the easiest and the most accessible technique. Microarray gene-expression analysis is a fast and accurate technique, but it is expensive, cumbersome, and not accessible in clinical laboratories. As a result, real-time PCR expression analysis of a molecular marker might be a more feasible method in routine diagnostic laboratories.

NOTCH1 is one of the main participants in Notch signaling pathway which starts the pathway. Previous studies showed that the aberrant Notch signaling had tumor-promoting function in breast cancer (Mittal et al. 2009).

Herein, we performed real-time PCR to relatively quantify the changes in NOTCH1 expression at mRNA level in breast cancer clinical samples. First, we divided patient into two subgroups based on histopathological reports; patients diagnosed with IDC and patients diagnosed to have other types of breast cancer. Gene expression analysis showed that the expression of NOTCH1 in IDC patients were increased dramatically compared to other histopathological types. Therefore, high-level expression of NOTCH1 in breast cancer can be used as a prognostic marker for detecting IDC. In addition, we found a slight correlation between over expression of NOTCH1 and p53 gene. Furthermore, previous IHC reports showed that mutated p53 protein was accumulated in the nucleus of tumor cells. Patnayak et al. 2015, retrospectively investigated 389 cases of breast cancers. They found no correlation between hormone markers, but they reported over expression of p53 in invasive breast cancer (Patnayak et al. 2015). Kim et al. 2015, tested 119 invasive ductal carcinoma samples and proposed ER as a marker of relapse and metastasis to axillary lymph nodes in invasive breast cancer (Kim et al. 2015), while in our limited sample size, we found no correlation between hormone receptors and the status of disease.

Hence, NOTCH1 and p53 seem to be a precious indicator of ductal carcinoma type for patients with breast cancer.

However, accumulated studies have shown alterations in gene expression of breast tumor cells as biomarkers for predicting prognosis outcome, mostly with contradictory results (van ‘t Veer et al. 2002). Therefore, it is most likely that using one gene has limited the predictive value, and such approaches with a combination of genes evaluated on more clinical samples are needed.
